# International Network for the Availability of Scientific Publications: Facilitating Scientific Publishing in Developing Countries

**DOI:** 10.1371/journal.pbio.0020326

**Published:** 2004-11-16

**Authors:** Pippa Smart

## Abstract

The International Network for the Availability of Scientific Publications (INASP) was established in 1992 to bridge the information divide between the developed and developing world

*‘The most important element that restricts our researchers is access to information.’—Subbiah Arunachalam, India, 2003*

The International Network for the Availability of Scientific Publications (INASP) was established by the International Council for Science in 1992 to provide support for networking between information providers and users, particularly to bridge the information divide between the developed and developing world. Since 1992 INASP has worked, in response to requests, to develop its activities for capacity building in information production, access, and use, with an overarching vision that all people are able to access and contribute information, ideas, and knowledge necessary for sustainable and equitable development.[Fig pbio-0020326-g001]

**Figure pbio-0020326-g001:**
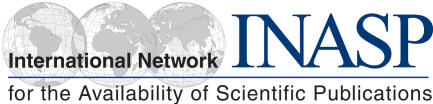


To ensure that INASP activities are appropriate for the communities and cultures of the countries in which INASP works, local partners are used to build networks and to provide advice and support. Enhancing capacities is central to all activities, and local ownership and sensitivity to local conditions and opinions are of paramount importance. Here I highlight two aspects of our work: increasing access to information and supporting the visibility of regional journals within the global research community.

## Programme for the Enhancement of Research Information

The growth and acceptability of the internet during the 1990s opened up tremendous possibilities for the dissemination of research information, providing many nations with access to information that had previously been out of their reach. Although internet connectivity remains problematic in many countries, it still offers great potential for bridging the information gap.

Arising from discussions with librarians and researchers in 1999 and 2000, the Programme for the Enhancement of Research Information was formally launched in 2002 after a two-year pilot programme. The Programme for the Enhancement of Research Information works in a two-phase manner: (1) providing and supporting access to international research information and (2) supporting and promoting access to nationally published research.

To support access to international information, INASP negotiates for heavily discounted or free access to online information from publishers and information providers in developed countries. Enabling access, however, does not guarantee that resources are used; both training and promotion are needed so that researchers and downstream information providers know how to make the best use of what is available. With this in mind, INASP has set up four series of training workshops, which have trained over 1,000 people from over 200 institutions in over 17 countries during the last two years. Although some of the training is undertaken by INASP staff, most is facilitated by in-country trainers.

Planning for long-term sustainability, INASP aims to hand over the tasks of negotiation, purchasing, and training to local consortia, associations, or networks.

## African Journals OnLine

Although much needed, access to international resources can discriminate against nationally published scholarly information. This may be due to one of the following: a perception that local publications are lower quality, distribution problems and irregular publishing, or lack of online visibility. However, national publications provide vital access to potential collaborators and information about research on topics of local relevance—since 1998, INASP has provided support to indigenous research publications to enable them to survive and coexist with international information.

One specific project that exemplifies these aims is known as African Journals OnLine (AJOL). AJOL launched in 1998 in response to requests from African journals. Starting with only ten titles, it now includes 189 from 21 African countries. Access to the site, which includes tables of contents, article abstracts, and a homepage for each journal with information about editorial boards, guidelines for authors, and more, is entirely free. Participating journals report increased international submissions and increasing contact with international researchers.

Researchers make wide use of this service, and over 8,000 people have registered to use AJOL since it launched. Registration is optional and until March this year did not provide the user any benefit—it simply provided an indication of the number of people visiting and from which countries. However, since March 2004 anyone who registers can sign up to receive a free E-mail table of contents alert. Over 500 people have chosen to receive E-mail alerts from an average of four journals each, and the effect of these alerts is felt through the increase in document delivery requests.

AJOL does not currently host full text, but can provide full-text articles through a document delivery service (there are plans to load full-text articles on AJOL in the future). This service is provided to researchers from developing countries for free and to researchers elsewhere at a minimal charge (to cover costs, plus a small payment to the journal). In the first six months of 2004, over 700 articles were ordered and sent out.

Most journals publishing out of Africa are not run by commercial publishers, and most of them operate at a loss, subsidised by universities, associations, funding agencies, or a mixture of all three. Paid subscriptions to print journals are frequently a vital part of their economics, and the financial viability of many journals is constantly under threat. Originally, it was hoped that AJOL would increase subscriptions, thereby providing greater financial security to the journals. This has not been achieved and is unlikely to occur in the future. However, with increasing visibility, the value of the journals increases along with, hopefully, their importance to the supporting agencies and long-term sustainability.

Since AJOL is set up to provide support for the participating journals, the journals' opinions are constantly sought before any developments are undertaken, meaning AJOL is effectively “owned” by them. Although the service is not actively run by the journals themselves, all participating journals are considered to be part of a community, receiving regular E-mail contact and skills support. Many journal editors (who frequently undertake all the publishing activities) feel isolated and unskilled, and even though AJOL does not operate as an association for the journals, it encourages communication between the editors, and a sharing of experience.

The AJOL website was relaunched in March 2004 using open-source software called Open Journal System, originally developed in Canada by the Public Knowledge Project at the University of British Columbia and further developed for AJOL at Bristol University, United Kingdom. This software was written to enable a single journal to manage the entire publishing process online from submission to publishing. Being open source, the software can also easily be adapted and modified. The Public Knowledge Project set up the system with the developing world in mind: it operates efficiently at low bandwidths and is easy to use, with many guides and help functions built in.

For the users, this software offers sophisticated searching, E-mail alerts, and a space for each journal within AJOL for journal-specific information. It also now provides a range of management tools, which makes the service more efficient and enables it to grow.

Another important consideration for choosing this system was that individual journals can take over the responsibility for maintaining their own journal areas within AJOL via the Internet. Over 50 journals have expressed an interest in taking this on, and one publisher is already successfully maintaining its material. To support this, training workshops are currently in preparation. This development will assist the long-term sustainability of AJOL and also give the participating journals experience in managing and publishing content online.

In the near future, INASP will be including full text on AJOL and hopes to move the management of AJOL to an African organisation so that it will truly become an African gateway for published research. In addition, INASP continues to work with journals to strengthen their quality and sustainability, providing advice, training workshops, study tours, and a coordinating point for discussion and collaboration.

Outside Africa, INASP is working on similar developments in Nepal and the Caribbean and has received expressions of interest from Bangladesh, Sri Lanka, and Vietnam.

## The Future of Journal Access

It is vital that researchers everywhere in the world have access to reliable, relevant information. At the same time, providing access to international literature needs to be balanced with supporting local publications to ensure that indigenous knowledge is not lost, but can take its place in the research community and contribute to the continuing development of science. Worldwide access to information is central to all INASP activities, and INASP supports policies and activities that work towards this.

## Where to Find Out More

INASP: http://www.inasp.info

AJOL: http://www.ajol.info

Public Knowledge Project: http://pkp.ubc.ca

## Abbreviations

AJOL: African Journals OnLine
INASP: International Network for the Availability of Scientific Publications

